# Treatment of IDH-mutant glioma in the INDIGO era

**DOI:** 10.1038/s41698-024-00646-2

**Published:** 2024-07-19

**Authors:** Mathew D. Lin, Alexander C.-Y. Tsai, Kalil G. Abdullah, Samuel K. McBrayer, Diana D. Shi

**Affiliations:** 1 Department of Medical Oncology, Dana-Farber Cancer Institute, Boston, MA 02215 USA; 2 Department of Neurosurgery, University of Pittsburgh School of Medicine, Pittsburgh, PA 15213 USA; 3 Hillman Comprehensive Cancer Center, University of Pittsburgh Medical Center, Pittsburgh, PA 15213 USA; 4 Children’s Medical Center Research Institute, University of Texas Southwestern Medical Center, Dallas, TX 75390 USA; 5 Department of Pediatrics, University of Texas Southwestern Medical Center, Dallas, TX 75390 USA; 6 Department of Radiation Oncology, Dana-Farber/Brigham and Women’s Cancer Center, Boston, MA 02215 USA

**Keywords:** CNS cancer, CNS cancer

## Abstract

Gliomas are the most common primary brain tumor and are uniformly lethal. Despite significant advancements in understanding the genetic landscape of gliomas, standard-of-care has remained largely unchanged. Subsets of gliomas are defined by gain-of-function mutations in the metabolic genes encoding isocitrate dehydrogenase (IDH). Efforts to exploit mutant IDH activity and/or directly inhibit it with mutant IDH inhibitors have been the focus of over a decade of research. The recently published INDIGO trial, demonstrating the benefit of the mutant IDH inhibitor vorasidenib in patients with low-grade IDH-mutant gliomas, introduces a new era of precision medicine in brain tumors that is poised to change standard-of-care. In this review, we highlight and contextualize the results of the INDIGO trial and introduce key questions whose answers will guide how mutant IDH inhibitors may be used in the clinic. We discuss possible combination therapies with mutant IDH inhibition and future directions for clinical and translational research.

Gliomas are the most common primary malignant brain tumor. Until recently, glioma subclasses had been exclusively defined based on histological subtype and grade (2–4)^1^. In the past two decades, our understanding of glioma biology has deepened substantially, driven largely by the discovery of predictive and prognostic mutations recurrently observed in gliomas. Mutations in genes encoding isocitrate dehydrogenase (IDH) metabolic enzymes have been described for well over a decade^2^ and are now formally incorporated into the World Health Organization diagnostic criteria for brain tumors^3^. *IDH* mutations are a defining feature of oligodendrogliomas and subtypes of astrocytoma, with the additional presence of 1p19q chromosomal codeletion further distinguishing oligodendrogliomas (1p/19q codeleted) from IDH-mutant astrocytomas (1p/19q intact).

Wild-type IDH enzymes exist in three isoforms, IDH1, IDH2, and IDH3^4,5^. All isoforms convert isocitrate to 2-oxoglutarate (2OG), using either NADP^+^ (IDH1/2) or NAD^+^(IDH3) as a cofactor. The conversion of isocitrate to 2OG is a critical step in the Krebs cycle and additionally serves as a cellular source of NADPH/NADH. 2OG also serves as a substrate for families of 2OG-dependent enzymes, including dioxygenases, dehydrogenases, and transaminases^4^. The majority of *IDH* mutations in gliomas affect *IDH1*, the most common of which results in an arginine to histidine substitution (IDH1-R132H)^6^. The mutant IDH1 (mIDH1) IDH1-R132H protein converts 2OG to the oncometabolite (*R*)-2-hydroxyglutarate [(*R*)-2HG)]^4,6^. Due to the structural similarity between (*R*)-2HG and 2OG, (*R*)*-*2HG competitively inhibits 2OG-dependent enzymes, including 2OG-dependent dioxygenases that affect DNA and histone methylation. Indeed, IDH-mutant gliomas exhibit a distinct hypermethylation pattern called the glioma CpG island methylator phenotype (G-CIMP)^7–9^, underscoring the unique epigenotype of these tumors.

Standard-of-care treatment for IDH-mutant gliomas involves chemotherapy regimens and local therapies that have been used for over 20 years. The recently published INDIGO (Investigating Vorasidenib in Glioma) trial^10^, demonstrating a clinical benefit from the mIDH1/2 inhibitor vorasidenib, marks a forthcoming shift in this paradigm. In this review, we outline current standard-of-care, examine the INDIGO trial and its implications, and discuss clinical and translational questions that are critical next steps to applying results from INDIGO to management of patients with IDH-mutant glioma.

## Current standard-of-care

Standard care for IDH-mutant glioma incorporates surgery, radiation (RT), and/or chemotherapy. Treatment begins with maximal safe resection, which reduces symptoms due to mass effect and allows for tissue sampling for molecular and histopathological analysis^11–13^. Extent of resection is also thought to be prognostic, with increased extent of resection demonstrating an association with improved overall survival^12–16^. However, due to the highly diffuse nature of gliomas (both IDH-mutant and IDH-wild-type), adjuvant treatment is often required^6,17,18^.

Among patients with grade 2 IDH-mutant gliomas, adjuvant treatment is determined by risk group, with low-risk patients typically being defined as age ≤40 with gross total resection^19,20^. Patients deemed low-risk may undergo a “watch and wait” approach, where RT and chemotherapy can be deferred until tumor progression occurs^6,21^, while high-risk patients (age >40 and/or subtotal resection) often receive adjuvant RT with chemotherapy (PCV [procarbazine, lomustine, and vincristine] or TMZ), though in select cases may be candidates for “watch and wait”^19,20,22,23^.

For patients with grade 3 IDH-mutant glioma, adjuvant therapy with RT followed by TMZ or PCV is generally used for all patients. Institutional practice varies regarding choice of TMZ or PCV due to (1) an often greater toxicity profile with PCV and (2) lack of modern randomized clinical trial data directly comparing efficacy of TMZ vs. PCV in this setting. Currently, 1p/19q status often informs adjuvant treatment regimen choice in grade 3 IDH-mutant gliomas, with 1p/19q codeleted patients receiving RT + PCV and 1p/19q noncodeleted patients receiving RT + TMZ^6,18,24–26^. This is supported by recent data from the French POLA network demonstrating that among patients with grade 3 oligodendroglioma, RT + PCV was associated with significantly improved 5-year and 10-year overall survival compared to RT + TMZ^27^. The ongoing and redesigned^28^ CODEL trial directly compares adjuvant RT + TMZ and RT + PCV among patients with grade 2–3 oligodendroglioma and will provide further insight into this question. For patients with grade 3 IDH-mutant astrocytoma, data from the CATNON trial support use of RT and adjuvant TMZ^29^.

Given concerns regarding potential long-term toxicities from RT and a desire to reserve RT as an effective salvage option, there is ongoing interest in whether chemotherapy alone (without RT) can be used as upfront treatment in select patients. The ongoing POLCA (NCT02444000) and NOA-18 trials (NCT05331521) are exploring the omission of RT, using PCV (POLCA) or CCNU + TMZ (NOA-18) without concurrent RT.

Following inevitable tumor progression, the options for salvage therapy are largely the same: surgery, RT, and alkylating chemotherapy. As in the upfront setting, each of these therapies have short and long-term toxicity profiles, the latter of which are particularly relevant in this disease where many patients are young at diagnosis and survive long enough to experience long-term toxicities. Importantly, the long disease course of low-grade gliomas can render it challenging to study long-term toxicities from RT. Severity of such toxicities following use of modern radiation techniques is not well-characterized and warrants additional study.

## The INDIGO trial

### Early-phase studies and the INDIGO trial

Optimal upfront treatment strategies for IDH-mutant glioma have largely centered on use and sequence of the above discussed modalities: surgery, RT, and/or alkylating chemotherapy. While targeted therapies have become standard-of-care in other molecularly-defined cancers (such as EGFR-mutant lung cancers, HER2-positive cancers, and even other IDH-mutant cancers), drugs that target supposed driver mutations in glioma had not demonstrated clinically significant benefits until recently. Indeed, ivosidenib and enasidenib, inhibitors of mIDH1 and mIDH2, respectively, have been approved by the United States Food and Drug Administration for treatment of either IDH-mutant leukemias (enasidenib, ivosidenib) and/or IDH-mutant cholangiocarcinomas (ivosidenib), raising the question as to whether mIDH inhibitors may also demonstrate efficacy in IDH-mutant gliomas.

As such, multiple prospective clinical trials have tested mIDH inhibitors in gliomas. Response assessment in these studies was performed according to Response Assessment in Neuro-Oncology (RANO) criteria, with contrast-enhancing tumors utilizing RANO high-grade glioma guidelines^30^ and non-enhancing tumors using RANO low-grade glioma guidelines^31^. Ivosidenib, a selective mIDH1 inhibitor, was tested in a phase I trial that enrolled IDH1-mutant glioma patients who had recurred or not responded to standard-of-care therapy^32^. Although this phase I study is not powered for efficacy, disease control outcomes were reported as secondary endpoints. Patients with contrast-enhancing tumors (associated with more aggressive disease) had a 0% response rate (0/31) with 54.8% progressing on treatment. In contrast, among patients with non-enhancing tumors, one partial response (1/35, 2.9%) and an overall higher rate of stable disease (30/35, 85.7%) were observed. However, inferring efficacy of mIDH inhibition from these data are limited by the fact that ivosidenib has modest blood-brain barrier penetration^33^. Subsequent trials have since tested the dual mIDH1/2 inhibitor vorasidenib^34^, which is more CNS-penetrant and exhibits a significantly higher tumor:plasma ratio in IDH-mutant glioma patients compared to ivosidenib (1.69 vs. 0.10)^35^. Like ivosidenib, vorasidenib was also first tested in a phase I trial that enrolled patients with recurrent or progressive IDH-mutant gliomas^36^. While patients with contrast-enhancing disease still responded poorly to vorasidenib (0% response rate and 40% with progressive disease), patients with non-enhancing gliomas exhibited 18.2% (4/22) response rate and 72.7% (16/22) stable disease. When compared directly within the same phase I perioperative study^35^, vorasidenib demonstrated less variable 2HG suppression compared to ivosidenib and was selected for subsequent phase III testing.

Taken together, the early-phase trials suggested that mIDH inhibitors may be best employed in the earlier, more indolent disease setting, and that the role of mIDH inhibitor monotherapy among patients with advanced, contrast-enhancing disease is limited. The lack of efficacy in the contrast-enhancing disease setting may be due to acquisition of additional drivers in more advanced disease, and/or a “hit-and-run” effect of mIDH^37^. Nevertheless, these trials set the stage for the INDIGO trial, which aimed to test efficacy of vorasidenib in upfront treatment of low-grade gliomas. The INDIGO trial enrolled patients with residual or recurrent grade 2 IDH-mutant oligodendroglioma or astrocytoma, who had not had any prior therapy other than surgery (1–5 years prior to randomization)^10^. Residual/recurrent disease was defined as ≥1 target lesion measuring ≥1 cm by ≥1 cm in longest dimensions. Importantly, patients were asymptomatic, not being treated with steroids for symptoms due to glioma, and had either minimal or nonnodular enhancement on magnetic resonance imaging (MRI) scans. Patients were randomized to vorasidenib or placebo control. The primary endpoint of the trial was progression-free survival (PFS), with a secondary endpoint being time to next intervention, defined as initiation of next anticancer treatment.

At a median follow-up of 14.2 months, median PFS was significantly improved with vorasidenib (27.7 months) compared to placebo (11.1 months) (hazard ratio [HR] 0.39, 95% CI 0.27–0.56, *p* < 0.001). Time to next intervention was also significantly improved with vorasidenib (HR 0.26, 95% CI 0.15–0.43, *p* < 0.001). Toxicities were mild, with grade ≥3 elevations in alanine aminotransferase (ALT) observed in 9.6% of patients receiving vorasidenib and 0% patients receiving placebo. Frequency of seizures was comparable between vorasidenib and placebo arms, with no clinically meaningful differences in patient-reported health-related quality of life^38^.

The INDIGO trial represents a step forward in advancing the treatment of IDH-mutant gliomas. In a disease where systemic therapy regimens largely rely on alkylating agent chemotherapies that lack tumor specificity, the INDIGO trial is remarkable in its use of a molecularly-guided targeted therapy and helps transition glioma treatment into the era of precision medicine. Furthermore, the results from INDIGO also clearly demonstrate the role of mIDH and (*R*)-2HG as drivers in low-grade, non-enhancing IDH-mutant gliomas. These results introduce a new treatment paradigm in which candidates for a “watch and wait” strategy in the pre-INDIGO era (and perhaps others beyond these criteria) may now be considered for mIDH inhibitor monotherapy.

With this said, it is important to acknowledge the limitations of the current data. First, it will be important to clarify whether the current PFS benefit translates to meaningful differences in survival outcomes. Survival data may be difficult to interpret given that 31.9% of patients in the placebo arm crossed over to vorasidenib treatment at progression. If no survival benefit is observed in the vorasidenib arm, it will be difficult to know whether this is a true lack of effect, or whether this is confounded by crossover. In addition, it is not known the extent to which second-line therapies may effectively salvage patients who progress in the control arm. In the absence of survival data, deferring potential toxicities of salvage treatment (including RT) is arguably a clinically meaningful endpoint in and of itself^39^, though there is a lack of data quantifying the degree of toxicities from brain-directed RT in the modern era. Extrapolating toxicities of RT employed for other brain tumors^40–42^ is confounded by different dose regimens, treatment fields, and patient demographics. Second, the INDIGO trial demonstrated a PFS benefit of vorasidenib compared to patients treated with placebo, though it is unknown how efficacy of vorasidenib compares to RT + PCV or RT + TMZ, which is often used adjuvantly as standard-of-care among patients who do not undergo observation after surgery^6,18^.

### Other mutant IDH inhibitors

In addition to vorasidenib and ivosidenib, additional mIDH inhibitors have demonstrated ability to reduce brain tumor levels of 2HG and may be similarly studied in future phase 3 trials. The brain-penetrant mIDH1 inhibitor olutasidenib showed stable disease as a best response in 40% of recurrent/progressive IDH1-mutant glioma patients in a phase Ib/II trial^43^. Results from an ongoing phase I clinical trial investigating safusidenib (DS-1001b), another brain-penetrant mIDH1 inhibitor, demonstrated objective response rates of 17.1% and 33.3% in contrast-enhancing and non-enhancing tumors, respectively^44^. Safusidenib is currently undergoing additional clinical testing in the upfront setting (NCT04458272) and in the recurrent/progressive setting (NCT05303519). The mIDH1 inhibitor BAY1436032^45^ was well-tolerated and demonstrated a response rate (complete or partial) of 11.4% in patients with IDH-mutant low-grade glioma in a phase I trial^46^. Further randomized clinical trials may lead to inclusion of these drugs as additional options to treat patients with IDH-mutant glioma.

### Response heterogeneity and resistance to mutant IDH inhibition

As with any impactful clinical study, the INDIGO trial prompts many follow-up questions that will shape how mIDH inhibitors are used to treat gliomas. First, who should receive mIDH inhibitor therapy? The INDIGO trial enrolled a highly selected patient population with a very favorable disease profile: non-enhancing, grade 2 tumors (52.0% with 1p/19q codeletion) with no prior RT or chemotherapy and who would have otherwise been candidates for “watch and wait.” However, it is unclear whether patients who do not meet these narrow eligibility criteria will also benefit from mIDH inhibitors. This is especially relevant given that mIDH inhibition is clearly not an efficacious treatment strategy in all IDH-mutant glioma patients. Patients with aggressive, contrast-enhancing disease do not appear to benefit from vorasidenib^36^. How should mIDH inhibitors be used in the many patients whose disease risk profile falls between INDIGO enrollment criteria and contrast-enhancing disease? What clinical or molecular biomarkers may help identify patients who are most likely to respond and benefit from mIDH inhibitors? Existing clinical data suggest that grade 2 and grade 3 patients may have similar clinical outcomes, as evidenced by Reuss et al., who reported median overall survival times for patients with grade 2 and grade 3 IDH-mutant glioma across three separate clinical cohorts as 10.9 years and 9.3 years, respectively^47^. This observation was also corroborated in an independent Swedish cohort of patients, where comparable overall survival was observed among grade 2 and grade 3 IDH-mutant glioma patients without known *CDKN2A/B* deletions (11.4 years and 10.9 years, respectively)^48^. These data may support use of mIDH inhibitors in patients with grade 3 disease, though it is difficult to assess in the absence of randomized clinical data. Whether comparable survival between patients with grade 2 and grade 3 IDH-mutant tumors will translate to similar clinical benefit from mIDH inhibitor therapy is unknown. Furthermore, it is unclear whether vorasidenib monotherapy improves outcomes compared to adjuvant chemotherapy and RT, or whether combining mIDH inhibitors with adjuvant RT+chemotherapy would be superior to RT+chemotherapy alone.

Part of the difficulty in identifying patients who will respond to mIDH inhibitor treatment stems from the fact that it is unknown which of the many downstream effects of mIDH are reversed with mIDH inhibition, whether that reversibility changes over the course of the glioma life cycle, and whether additional driver mutations predominate in later stage disease. Few preclinical models exist that can be used to study oncogenic mechanisms of *IDH* mutations in the context of grade 2–3 disease^49,50^. The indolent nature of low-grade IDH-mutant gliomas renders these tumors difficult to generate patient-derived cell lines that proliferate in vitro or in xenograft models, and engineered approaches that utilize exogenous mutant IDH expression are limited in their ability to fully capture the genetic and epigenetic landscape observed in human tumors. As the microenvironmental effects of mutant IDH are becoming more understood, it also increases the importance and presents challenges for researchers to develop in vivo models generated in immune-intact (ideally, native immune) systems. Recent advances such as patient-derived organoids^51^, genetically engineered models of IDH-mutant glioma^52–55^, and assessment of samples acquired from patients treated with mIDH inhibitors^56^ may be helpful in this regard. Analysis from patients in the INDIGO trial may also provide insight in identifying molecular changes that correlate with response or resistance to vorasidenib.

**Fig. 1:**
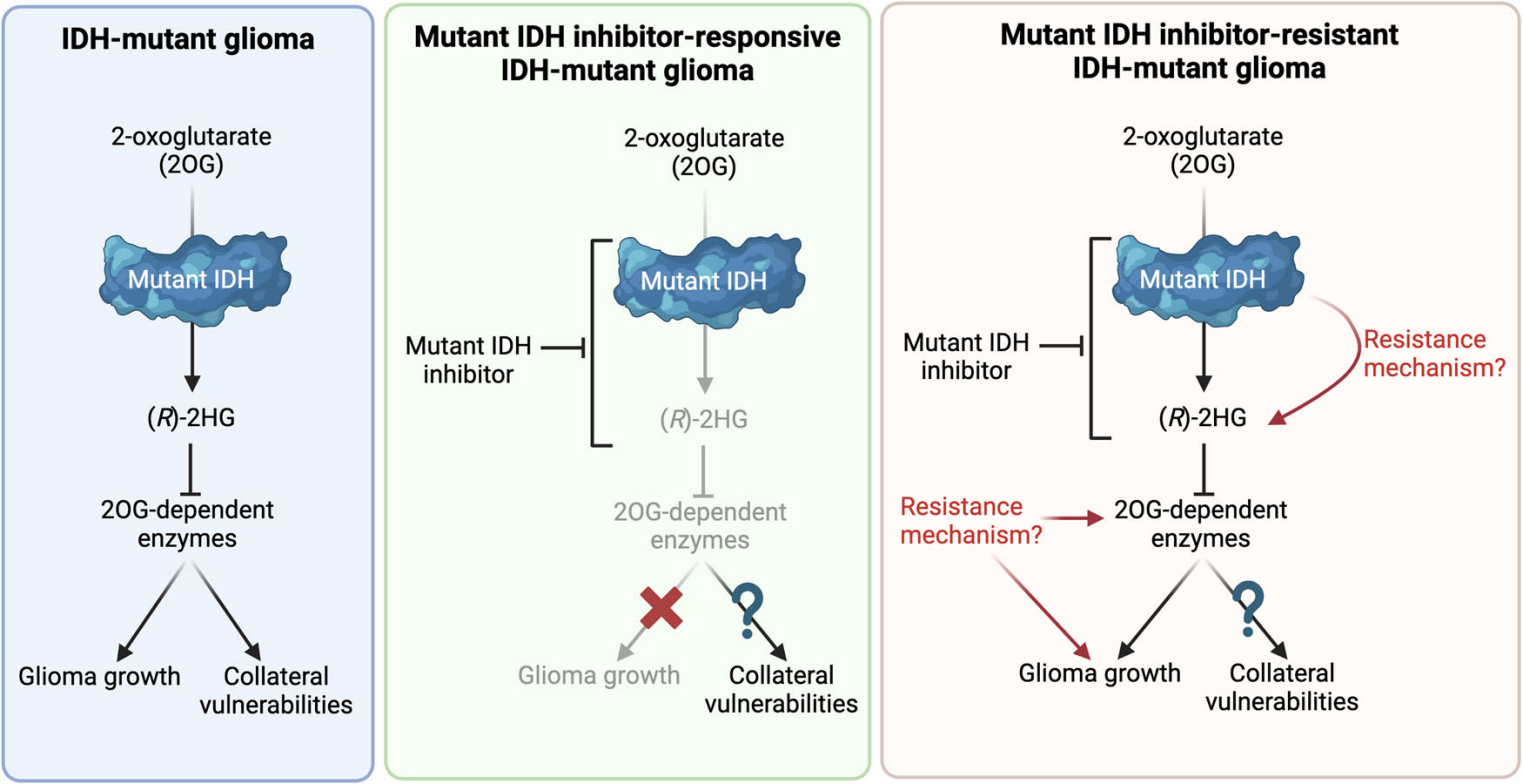
IDH-mutant gliomas and response to vorasidenib. Left: Role of mutant IDH in untreated glioma tumors. Collateral vulnerabilities include druggable synthetic lethal targets of mutant IDH. Middle: Schematic of mutant IDH inhibitor treatment in IDH-mutant gliomas responsive to mutant IDH inhibition. It is unknown whether strategies that target collateral vulnerabilities conferred by mutant IDH may still be utilized effectively in these settings. Right: IDH-mutant gliomas with de novo or acquired resistance to mutant IDH inhibitors. As in mutant IDH inhibitor-sensitive IDH-mutant gliomas, tumors resistant to mutant IDH inhibition may be candidates for therapies that target collateral vulnerabilities, depending on the mechanism of action.

A related question to understanding mechanisms of sensitivity is how IDH-mutant gliomas acquire resistance to mIDH inhibition (Fig. 1). Given the 28% progression rate reported in vorasidenib-treated patients enrolled in INDIGO, clinicians will increasingly need to decide how to employ salvage therapy for patients who have progressed on mIDH inhibitors. Part of this challenge is a lack of current understanding as to how prolonged mIDH inhibition alters the biology of IDH-mutant gliomas by time of progression. Do patients progress while on vorasidenib with high (*R*)-2HG, rendering them potential candidates for synthetic lethal strategies that exploit ongoing presence of (*R*)-2HG? Or do patients progress on vorasidenib with low (*R*)-2HG, indicating that salvage combination treatments that do not rely on elevated (*R*)-2HG for efficacy should be prioritized? It may also be the case that tumors progress during vorasidenib treatment with low (*R*)-2HG, but revert to a high (*R*)-2HG state upon cessation of mIDH inhibitor treatment. If so, then the kinetics of (*R*)-2HG re-accumulation in this setting are especially important in dictating choice and timing of salvage therapies. Given associations between *IDH* mutations and DNA damage deficits^57^, how do (*R*)-2HG levels impact efficacy of common salvage therapies such as RT?

Understanding these mechanisms of resistance are not only important for guidance of existing salvage treatment options, but may also reveal novel druggable targets for second-line therapies. If acquired resistance is driven by secondary *IDH* mutations that allow tumors to maintain (*R*)-2HG production (Fig. 1), drugs that block canonical and acquired *IDH* mutations may be beneficial, akin to secondary T790M EGFR mutations observed following tyrosine kinase inhibitor (TKI) treatment in EGFR-mutant lung cancer^58^. Such “(*R*)-2HG-restoring” mechanisms of resistance have been described in IDH-mutant leukemia patients treated with ivosidenib or enasidenib and in IDH-mutant cholangiocarcinomas^59–64^. However, it is also possible that resistance to mIDH inhibition in glioma may be mediated through (*R*)-2HG-independent mechanisms. Prolonged mIDH inhibition may select for silencing or activation of one or more downstream (*R*)-2HG effectors, akin to MET-amplification as an alternative mechanism of resistance to TKIs in EGFR-mutant lung cancer^65^. These are critical questions to address, particularly as the population of mIDH inhibitor-resistant IDH-mutant glioma patients is expected to substantially increase as vorasidenib (and perhaps other mIDH inhibitors) becomes increasingly utilized in the clinic.

## Additional emerging treatment strategies for IDH-mutant gliomas

Unfortunately, despite resection and standard-of-care chemotherapy and RT, progression is inevitable, which is likely to remain true even as longer-term data from the INDIGO trial are reported. Several ongoing efforts to translate novel therapeutic strategies from preclinical work are outlined below and continue to be relevant. There is additionally a pressing need to understand how these therapies may be best employed either with concurrent mIDH inhibitor treatment, or as salvage in the mIDH inhibitor-resistant setting.

## PARP inhibitors

(*R*)-2HG has been shown to impair homology-dependent DNA repair due to inhibition of 2OG-dependent enzymes involved in homologous recombination^66,67^. *IDH* mutations are thus thought to confer a “BRCA-like” defect that renders IDH-mutant tumors sensitive to poly(ADP-ribose) polymerase (PARP) inhibitors. Of note, this mechanistic model has been recently challenged by data suggesting that sensitivity of IDH-mutant tumors to PARP inhibition may instead be due to mIDH-dependent heterochromatin formation and resultant replication stress^68^. Sensitivity to PARP inhibitors may be further enhanced in IDH-mutant gliomas by impairments in NAD^+^ metabolism^69,70^. Currently, PARP inhibitors are being tested in early-phase clinical trials in IDH-mutant gliomas^6^. Results thus far from monotherapy testing in the “Using Olaparib in Recurrent IDH-mutant Glioma” (OLAGLI) trial have demonstrated limited success with olaparib alone^71^, prompting interest in results from ongoing trials using PARP inhibitors in combination with agents such as TMZ (NCT03914742) or immunotherapy (NCT03991832)^6^. Importantly, depletion of (*R*)-2HG with IDH inhibitor treatment reversed sensitivity to PARP inhibitors^67^, suggesting that PARP inhibitors may have decreased efficacy when used in combination with mIDH inhibitors or in tumors that progress on mIDH inhibitors with low (*R*)-2HG.

## CDK inhibitors

Homozygous deletion of the *CDKN2A/B* tumor suppressor confers a particularly poor prognosis in IDH-mutant gliomas^72^ and is now formally incorporated into WHO diagnostic criteria^3^. The p16INK4a protein encoded by *CDKN2A* normally functions to inhibit cyclin-dependent kinases 4 and 6 (CDK4/6). Loss of *CDKN2A* therefore increases CDK4/6 activity and can cause dysregulation in downstream targets of CDK4/6, including the tumor suppressor Rb. CDK4/6 inhibition is thus an appealing strategy in *CDKN2A/B*-deleted tumors and has been tested in other solid tumors with *CDKN2A* alterations^73,74^. Phase II trials are currently underway testing the CDK4/6 inhibitors palbociclib (NCT02530320) and abemaciclib (NCT03220646) in IDH-mutant oligodendrogliomas.

## Demethylating agents

Given that (*R*)-2HG competitively inhibits 2OG-dependent enzymes, many of which contribute to DNA and histone demethylation, there has been interest in whether methyltransferase inhibitors may be useful in treating IDH-mutant gliomas. Indeed, preclinical data show that the DNA methyltransferase inhibitor 5-azacytidine can reverse upregulation of the *PDGFRA* oncogene driven by *IDH* mutations^75^. One of the challenges associated with this strategy is that DNA hypermethylation patterns caused by mIDH^7–9^ include silencing of many genes, and it is not known (1) which of the locus-specific hypermethylation events are reversible, and (2) whether reversal of hypermethylation will be effective in halting tumor growth in patients, as it is in mouse models^76,77^. Results from ongoing trials testing the DNA methyltransferase inhibitors decitabine and 5-azacytidine in recurrent IDH-mutant gliomas (NCT03922555 and NCT03666559, respectively) will be helpful in this regard.

## Metabolic pathway inhibitors

Additional therapeutic strategies in various stages of clinical translation have leveraged the metabolic dependencies conferred by mIDH to selectively target IDH-mutant gliomas. Glutaminase inhibitors have been shown to exploit metabolic defects conferred by *IDH* oncogenes in glioma^78,79^. (*R*)-2HG inhibits 2OG-dependent branched chain transaminases BCAT1/2 in IDH-mutant gliomas, creating a dependency on glutaminase to maintain glutathione pools. Glutaminase inhibitors thus exploit this defect and effectively decrease glutathione pools in IDH-mutant gliomas, increasing sensitivity to RT and oxidative stress. A phase Ib trial using the glutaminase inhibitor teleglenastat (CB-839) in combination with RT in IDH-mutant grade 2–3 astrocytomas demonstrated acceptable safety profiles^80,81^. Importantly, redox stress induced by mIDH was rescued with mIDH inhibitor treatment^78^, suggesting that concurrent mIDH inhibitor treatment may antagonize teleglenastat/RT efficacy.

Work from Tateishi et al.^70^ and Nagashima et al.^82^ have similarly identified metabolic vulnerabilities conferred by mIDH that can be therapeutically targeted. Specifically, mIDH decreases NAD^+^ pools, creating a dependency that renders IDH-mutant gliomas sensitive to drugs that further deplete NAD^+^. Such strategies include inhibitors of nicotinamide phosphoribosyltransferase (NAMPT)^70^ or combination of poly(ADP-ribose) glycohydrolase (PARG) inhibition and TMZ^82^. Clinical studies of NAMPT inhibitors have been limited by toxicity^83,84^, but PARG inhibitors are currently in clinical development that could be tested for IDH-mutant glioma therapy in future trials.

More recently, several brain tumor types including IDH-mutant gliomas have been found to be sensitive to inhibitors of dihydroorotate dehydrogenase (DHODH), an enzyme in the de novo pyrimidine synthesis pathway^53,85,86^. IDH-mutant gliomas are hyperdependent on de novo pyrimidine synthesis, and treatment with the brain-penetrant DHODH inhibitor orludodstat (BAY2402234) induces replication stress and DNA damage^53^. Clinical trials are forthcoming to test orludodstat in IDH-mutant glioma patients.

## Immunotherapy

(*R*)-2HG has been shown to suppress the immune microenvironment through multiple mechanisms, including altered leukocyte chemotaxis^87^ and suppression of T cell function in both in IDH-mutant glioma^88–90^ and IDH-mutant cholangiocarcinoma^91^. These findings raise the question as to whether mIDH-driven immunosuppression can be reversed with mIDH inhibitor treatment, supported by data suggesting that immune activation by ivosidenib is a critical mechanism of action in IDH-mutant cholangiocarcinoma^91^. mIDH inhibitor treatment may therefore enhance efficacy of immune checkpoint blockade if used concurrently in IDH-mutant glioma, raising potential combination therapy strategies. Indeed, a phase II trial testing combination nivolumab and ivosidenib treatment in patients with advanced IDH-mutant solid tumors is underway (NCT04056910), as well as a phase I trial testing combination pembrolizumab and vorasidenib treatment in patients with grades 2 and 3 IDH-mutant glioma (NCT05484622).

In addition to immune checkpoint blockade, vaccines for both IDH-mutant and IDH-wild-type gliomas have presented exciting avenues for immunotherapy in brain tumors. A recent phase I clinical trial demonstrated promising results from a peptide vaccine specific for the IDH1-R132H mutant protein^92^. Among the 33 patients enrolled, 93.3% (30/32) displayed an immune response induced by the IDH1-R132H vaccine across multiple HLA alleles, with an overall response rate of 84.4% (per RANO criteria, including stable disease) and acceptable safety profiles. Because mIDH inhibitors have been shown to reverse (*R*)-2HG-mediated T cell suppression, it is plausible that they could augment immunogenicity of IDH1-R132H vaccines. This rational combination therapeutic strategy may be tested in future studies.

## Future clinical and translational directions

Results from the INDIGO trial are poised to establish a new standard in the treatment of IDH-mutant glioma, a significant advance in a field that is in desperate need of effective clinical therapies. IDH-mutant gliomas are the most recent IDH-mutant cancers to demonstrate clinical benefit from mIDH inhibitor treatment (following IDH-mutant leukemia and IDH-mutant cholangiocarcinoma), and these results reflect the culmination of more than a decade of basic, translational, and clinical research efforts. The INDIGO trial results also underscore the importance of several questions: (1) what are the molecular mechanisms that mediate sensitivity of gliomas to mIDH inhibition? (2) what biomarkers predict who will respond to mIDH inhibitors? (3) how do tumors acquire resistance after treatment with mIDH inhibitors? (4) how should mIDH inhibitors be used in combination with existing and emerging treatments for IDH-mutant gliomas?

Addressing these questions requires a deeper understanding of IDH-mutant glioma biology. Mechanisms underlying sensitivity and resistance to mIDH inhibitor treatment are likely to be closely related to the downstream oncogenic targets of mIDH, which are not fully understood. Thoughtful combination treatment strategies necessitate an understanding of how new and existing therapies for IDH-mutant glioma exert their efficacy, allowing for rational integration of these treatments with mIDH inhibitors. Additional clinical (and molecular) data from patients treated with mIDH inhibitors will also be useful in addressing these questions. In addition, preclinical data using glioma models responsive to mIDH inhibitor therapy will serve as important systems for mechanistic investigation. Collectively, these efforts will refine how mIDH inhibitors should be optimally used in order to maximize clinical benefit for IDH-mutant glioma patients.

## Data Availability

There are no original data presented in this review article. All data discussed in this article can be found in the References section.
